# Solubilities of Ethylene and Carbon Dioxide Gases in Lithium-Ion Battery Electrolyte

**DOI:** 10.1021/acs.jced.3c00692

**Published:** 2024-05-21

**Authors:** Mel Soto, Kae Fink, Christof Zweifel, Peter J. Weddle, Evan Walter Clark Spotte-Smith, Gabriel M. Veith, Kristin A. Persson, Andrew M. Colclasure, Bertrand J. Tremolet de Villers

**Affiliations:** † National Renewable Energy Laboratory (NREL), 15013 Denver West Parkway, Golden, Colorado 80401, United States; ‡ Department of Materials Science and Engineering, University of California, Berkeley, California 94720, United States; § Materials Science Division, Lawerence Berkeley National Laboratory, Berkeley, California 94720, United States; ∥ Chemical Sciences Division, Oak Ridge National Laboratory, Oak Ridge, Tennessee 37831, United States; ⊥ Molecular Foundry, Lawerence Berkeley National Laboratory, Berkeley, California 94720, United States

## Abstract

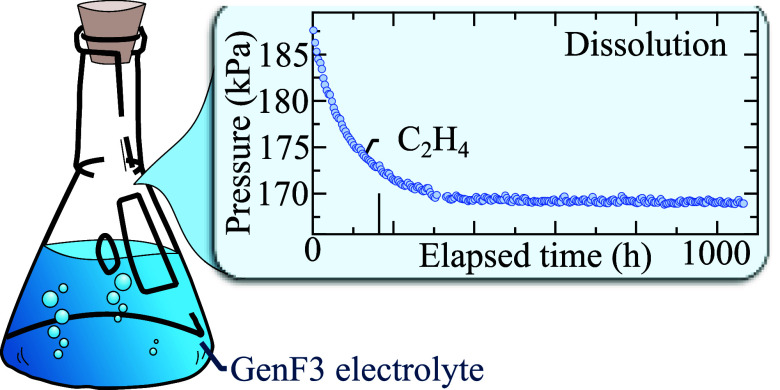

During Li-ion battery operation, (electro)chemical side reactions occur within the cell that can promote or degrade performance. These complex reactions produce byproducts in the solid, liquid, and gas phases. Studying byproducts in these three phases can help optimize battery lifetimes. To relate the measured gas-phase byproducts to species dissolved in the liquid-phase, equilibrium proprieties such as the Henry’s law constants are required. The present work implements a pressure decay experiment to determine the thermodynamic equilibrium concentrations between the gas and liquid phases for ethylene (C_2_H_4_) and carbon dioxide (CO_2_), which are two gases commonly produced in Li-ion batteries, with an electrolyte of 1.2 M LiPF_6_ in 3:7 wt/wt ethylene carbonate/ethyl methyl carbonate and 3 wt % fluoroethylene carbonate (15:25:57:3 wt % total composition). The experimentally measured pressure decay curve is fit to an analytical dissolution model and extrapolated to predict the final pressure at equilibrium. The relationship between the partial pressures and concentration of dissolved gas in electrolyte at equilibrium is then used to determine Henry’s law constants of  2.0 × 10^4^ kPa for C_2_H_4_ and *k*_CO_2__ = 1.1 × 10^4^ kPa for CO_2_. These values are compared to Henry’s law constants predicted from density functional theory and show good agreement within a factor of 3.

## Introduction

Li-ion batteries are currently one of the most energy-dense commercial battery chemistries, dominating the market for electronic devices, electric automotives, and stationary energy storage.^1−3^ However, further energy density improvements are required to electrify markets such as flight, freight, and maritime transport.^1−4^ In pursuit of next-generation batteries with even higher energy densities, new chemistries for anodes, cathodes, and electrolytes are continuously being investigated. At present, some of the materials receiving the most interest and scrutiny include silicon or lithium metal as replacements for graphite anodes,^5−9^ lithium-, manganese-, and nickel-rich cathodes,^10,11^ and ether-based localized high-concentration electrolytes.^12^ However, some of these materials have yet to achieve mass commercialization due to significant shortcomings in cycle and/or calendar lifetimes as a result of unstable reactivities between the electrodes and the electrolyte.

A stable, electronically passivating, and ion-permeable solid-electrolyte interphase (SEI) is a key feature of Li-ion batteries, as it protects the electrode from continuous side reactions with the electrolyte while enabling Li-ion transport. While various additives, usually liquids, have been explored to improve the stability and permeability of anode SEIs, relatively little research has been done to determine the role of gases in the performance of commercial Li-ion battery chemistries, let alone next-generation battery compositions.^5,13^ Gas generation and subsequent consumption at the anode SEI has been documented in nondegassed pouch cell systems.^14^ Many gases are generated during Li-ion battery cycling, any of which could have possible beneficial or adverse effects on performance. Early work on Li-metal anode and Li-graphite anode batteries explored CO_2_ as an additive, finding it improved cycling efficiency^15−18^ and stability of the graphite SEI.^15,19,20^ These beneficial consumptive effects of CO_2_ are typically associated with a more favorable SEI through the suppression of transesterification reactions and production of more cross-linked poly(ethylene oxide)-type polymeric species.^17,21^ More recently, Blaubaum et al. studied Li-ion batteries containing electrolyte saturated with CO_2_, CO, C_2_H_4_, C_2_H_2_, H_2_, CH_4_, and O_2_ (all gases that are commonly reported in Li-ion batteries), and concluded that battery electrolytes saturated with CO_2_ and O_2_ showed higher C-rate capabilities and less irreversible capacity loss during the first cycle.^13^

Gas-phase byproducts typically result from electrochemical oxidation/reduction of the electrolyte solvent species. In the electrolyte system evaluated in this work, fluoroethylene carbonate (FEC) has the highest reduction potential, and thus is most likely to decompose to form species such as vinylene carbonate and lithium fluoride (LiF) .^22,23^ Ideally, gas species formation could be used as a signature of a particular electrolyte species reduction, if the reaction mechanisms for electrolyte decomposition are known.^24^ For example, CO is typically attributed as a byproduct from the reduction of ethyl methyl carbonate (EMC) and subsequent transesterification to diethyl carbonate and dimethyl carbonate (DMC).^23,25,26^ As mentioned above, the presence of CO_2_ is generally reported to have favorable effects on the cycle life.^17,18,27^ However, competing pathways are sufficiently complex that attributing a particular gas-phase product to a certain liquid-phase reactant has proven difficult. When trying to untangle the influence of a particular species on reaction pathways, it is important to consider whether dissolved species are preferentially retained in the electrolyte or expelled away from the electrode into the reduction-free gas phase. For example, if the thermodynamics of CO_2_ enable greater stability as a gas-phase species compared to a dissolved liquid-phase species, reaction mechanisms involving CO_2_ consumption in the bulk, liquid electrolyte would be less likely.

To understand the availability of a particular gaseous species at the electrode interface to form the SEI, the gas species solubility in the electrolyte must be known. The solubility of nonpolar gases, including ethylene, in DMC has been studied previously by a gas saturation method measuring volume of gas dissolved into a known volume of solvent.^28,29^ Ethylene solubility in other common battery solvents such as ethylene carbonate (EC) and EMC has not yet been reported. Further, previous studies on CO_2_ solubility in common electrolyte solvent mixtures employed a method involving full gas saturation and subsequent displacement and chemical titration of the dissolved CO_2_.^30,31^ These studies indicate that the addition of Li salts such as LiPF_6_ can also increase the solubility of the gas, emphasizing the need to study real electrolyte systems. Each of these two methods were developed to study a specific subset of gas-phase species, but each also has notable limitations. In particular, the latter approach (chemical titration), which was used to study CO_2_ is poorly suited to detect nonpolar gases; the former method (measuring the volume of dissolved gas in solvent) is better-suited for nonpolar gases, but introduces temperature differences over the entire system that require correction factors. Thus, neither of these approaches is optimal to conduct solubility measurements for the practical application of battery electrolyte interfaces, where precise quantification of solubility across a broad class of gaseous species is required. Instead, a method for investigating solubility of both polar and nonpolar gases with a temperature-controlled closed system pressure differential technique is employed in this work. Solubility values are obtained by monitoring the pressure decay of a gas above a liquid at a constant temperature until a steady state is reached.^32,33^ The measured change in pressure corresponds to the concentration of gas in the saturated liquid at the final steady state.

The pressure decay technique often requires long time scales to reach equilibrium, especially in systems with high viscosity where the measurements take days to weeks.^32,33^ To improve experimental measurement throughput, the final equilibrium pressure *P*_eq_ can be extrapolated from initial data using a nonlinear regression based on the molar flux of diffusion outlined in Fick’s laws. To this end, an equation derived by Behzadfar and Hatzikiriakos^32^ has been used in this study to model the pressure decay of the system given initial pressure decay data to return *P*_eq_. Discussion of assumptions used in this model and the subsequent calculation of solubility is included in the pressure decay modeling section below.

We present an apparatus and associated methodology to determine the solubility of carbon dioxide and ethylene gases in a battery electrolyte by measuring the pressure change during dissolution of the gases into the liquid. The gaseous species concentration in the liquid has been plotted against the pressure of the gas above the liquid to determine the Henry’s law constant, *k*
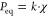
1where χ is the saturated gas solubility as a mole fraction and *k* is the Henry’s law constant in kPa.

## Experimental Section

Chemicals used in this study, their suppliers, and other relevant information are summarized in Table 1.

**Table 1:** Chemicals Used in this Study, Suppliers, Purity, and Purification Methods

chemical	supplier	initial purity, mol fraction	purification method	final purity, mol fraction	analysis method
15 wt % lithium hexafluorphosphate (LiPF_6_), 25.5 wt % EC, 59.5 wt % EMC^a^	Tomiyama Pure Chemical Ind., Ltd.	0.9998		0.9998	
FEC	Tomiyama Pure Chemical Ind., Ltd.	0.99		0.99	
carbon dioxide (CO_2_)	GASCO	0.9999		0.9999	
ethylene (C_2_H_4_)	Sigma-Aldrich	≥0.995		≥0.995	
water	Tap		Barnstead E-pure ultrapure water purification system	>0.9999	resistivity

a Formulation was purchased premixed by the supplier, and the uncertainty of the composition is unknown.

### Pressure Decay Trials

The pressure decay experiments were conducted in a custom-built stainless-steel Swagelok cell, modified from a design developed by Oak Ridge National Laboratory (Figure S1).^34^ The pressure was monitored using an Omega PX-409 USBH pressure transducer. At the start of each pressure-decay trial, the entire cell was evacuated to approximately 5.9 kPa, and then filled with the target gas to the desired pressure. In hard-cased silicon-based Li-ion batteries, internal pressures have been reported to vary from approximately 300–950 mbar relative to atmospheric pressure due to expansion and contraction during cycling.^35^ Considering a standard atmospheric pressure of 101 kPa, the absolute pressures of gas in the cell ranges from 131 to 196 kPa. Therefore, three filling pressures were chosen: 18, 28, and 40 psia, corresponding to target gas initial partial pressures ranging from 124 to 276 kPa. After gas filling, a needle valve was closed to trap gas in the gas reservoir. The other side of the cell was opened to allow the remaining gas to escape and remained open as the apparatus was cycled through an antechamber into a glovebox with argon atmosphere.

Inside the glovebox, 2 mL of electrolyte (comprising 15:25:57:3 wt % LiPF_6_/EC/EMC/FEC, hereafter referred as GenF3) was added into the electrolyte reservoir using a syringe with an 18 gauge, 8″ needle. The argon pressure in the box was recorded and a ball valve was closed to seal the cell. The cell was brought out of the glovebox and placed into a temperature-controlled chamber at 30 °C (303 K). The cell was allowed to rest for 2 h to allow the internal pressure to equilibrate. The equilibrated value was recorded as *P*_gr_ and indicates the pressure of the gas reservoir before the trial began. The needle valve was then fully opened to release the target gas into the electrolyte chamber. The pressure decay over time was recorded in 3 s intervals on the Omega Transducer software.

Accurate volume measurements of the entire cell were needed to calculate the moles of gas present in the system during the trials. This was done separately using a difference-in-mass method with deionized water. During volume measurements, the transducer was replaced by a solid steel NPT plug, which was tightened to the same depth as the transducer. The cell with all valves open was fully dried at 80 °C (353 K) overnight in a vacuum oven, then cooled and weighed empty. The cell was then filled with water through the electrolyte reservoir, capped, and then reweighed. This was repeated and the average weight was converted to volume using the density of water. The volumes for the gas and electrolyte chambers were determined by experiment. Briefly, the gas reservoir was filled with air, the needle valve was closed, and the electrolyte chamber was evacuated to approximately 5.9 kPa. The ball valve was closed to isolate the entire cell and the cell was placed in an oven to equilibrate at 30 °C. Following equilibration and recording of the gas reservoir initial pressure, *P*_gr_, the needle valve was opened to allow the gas on the gas reservoir side to expand and fill the vacuum. This final pressure at equilibrium was recorded. The ratio of the pressures before and after the release of the gas were used to calculate the volume ratio of the gas reservoir side to the entire cell volume.

To evaluate the capability of the measurement apparatus and test the validity of our data analysis methods (described in more detail in the next section), the Henry’s law constant of carbon dioxide in water at 30 °C (303 K) was measured, Figures S2 and S3. The experimental solubility value was found to be 1.92 × 10^5^ kPa; data is provided in Table S3. Carbon dioxide solubility in water has been extensively characterized with a reported Henry’s law constant of 1.85 × 10^5^ kPa at 303.15 K,^36^ suggesting that the present experimental method has a high degree of accuracy for prediction of Henry’s law constant values, Table S4.

### Pressure Decay Modeling

The pressure decay of a gas dissolving into a liquid through mass transfer was modeled by Behzadfar and Hatzikiriakos^32^ and results in the analytical expression given by
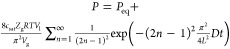
2where *P* is the measured pressure, *c*_sat_ is the saturation concentration in mole fraction, *Z*_g_ is the gas compressibility (0.99426 and 0.99 for C_2_H_4_ and CO_2_, respectively), *V*_l_ is the volume of the liquid (2.0 mL), *D* is the species liquid-phase diffusivity, *L* is the diffusion length (7.02 mm), *R* is the universal gas constant, *T* is the temperature, and *V*_g_ is the volume of the gas (12.78 mL), see also Table S1. Equation 2 solves for the pressure at time *t*, and notably does not model the initial pressure drop due to the interface filling phenomenon that occurs at the beginning of the experiment.^32^ To use this expression, our data sets exclude this early stage pressure drop to obtain a more accurate regression. It is assumed that the electrolyte, prepared only in the glovebox, has little to no C_2_H_4_ or CO_2_ gas dissolved it in to begin with. The data sets are fit to eq 2 by iteratively solving for the final pressure, *P*_eq_, and the diffusivity *D* that minimize the sum of squares of the error differences between the experimental and model data points (i.e., least-squares regression fitting).

After all trials at the same gas reservoir pressure had concluded and been modeled, their diffusivities were averaged and input back into eq 2. The fit was then determined by keeping *D* constant and only varying *P*_eq_. These final produced *P*_eq_ values were used for subsequent solubility calculations and the construction of the Henry’s law curve.

It is important to note that *c*_sat_ in the Behzadfar model is calculated from the first pressure point of the data used for regression and the solved variable *P*_eq_.^32^ As this necessarily excludes the early stage pressure drop and involves the total pressure, the real *c*_sat_ value was calculated separately for the Henry’s law graphs. The solubilities were calculated from the difference in target gas moles between the initial and equilibrium conditions. The target gas initial and equilibrium partial pressures were calculated by subtracting the partial pressure of argon from the glovebox above the electrolyte and the vapor pressure of a similar electrolyte at 25 °C (1 M LiPF_6_ in 3:7 wt/wt EC/EMC)^37^ from each trial’s *P*_eq_ value. These Ar and vapor pressures were assumed constant throughout the duration of each trial (Table S2).

To supplement and corroborate experimental measurements, the Henry’s Law constant was also predicted using density functional theory (DFT). All DFT calculations used the Q-Chem electronic structure code version 5^38^ with the ωB97X-V range-separated hybrid generalized gradient approximation exchange–correlation functional^39^ and the def2-TZVPPD basis set.^40^ Optimized structures, electronic energies (*E*), enthalpies (*H*), and entropies (*S*) corresponding to CO_2_ and C_2_H_4_ in solution were taken from the lithium ion battery electrolyte (LIBE) data set.^26^ In LIBE, solvent effects are treated implicitly using the solvent model with density (SMD),^41^ using parameters relevant to a 3:7 wt/wt mixture of EC and EMC. SMD is based on the polarizable continuum model,^42^ which models a bulk solvent environment as a uniform dielectric medium surrounding a solute-shaped cavity. In addition to the PCM-like bulk electrostatic terms, SMD adds energy terms accounting for short-range interactions, namely cavitation, dispersion, and local solvent structure. The solvent-optimized CO_2_ and C_2_H_4_ structures from LIBE were reoptimized in vacuum, and then the vacuum-optimized structures were subjected to a vibrational frequency analysis to obtain the gas-phase thermochemistry (e.g., H, S).

Molecular free energies are calculated from DFT as
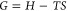
3where the enthalpy term (*H*) implicitly includes the electronic energy and zero-point energy. For this study, we consistently used *T* = 303.15 *K* = 30 °C, as our experimental measurements were taken at 30 °C.

The solvation free energy Δ*G*_solvation_ is calculated as
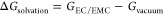
4where Δ*G*_EC/EMC_ is the free energy of the molecule in the solution phase (using SMD) and Δ*G*_vacuum_ is the free energy of the molecule in vacuum. From Δ*G*_solvation_, the Henry’s law coefficient *k* can be expressed^43,44^ as
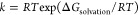
5

DFT calculations using SMD can predict the solvation free energies of small molecules with high accuracy. When calculating the solvation free energies of neutral molecules in one of the 90 nonaqueous solvents included in its training set, SMD achieves a mean unsigned error of 0.67 kcal mol^–1^ (0.03 eV).^41^ While one might reasonably expect a somewhat higher error when calculating solvation free energies in solvents outside of the training set, such as 3:7 wt/wt EC/EMC, the thermodynamics obtained from DFT should nonetheless be reasonably accurate for the types of small gases considered here. However, calculating Henry’s law coefficients is considerably more challenging as compared to computing the solvation free energies. As seen in eq 5, calculating the Henry’s law coefficient requires exponentiation of the solvation free energy, meaning even very small errors in Δ*G*_solvation_ can have a considerable impact on the predicted coefficient. As seen in eq 5, modifying Δ*G*_solvation_ by 1 kcal mol^–1^ would cause *k* to change by a factor of roughly 5.4.

## Results

**Figure 1:**
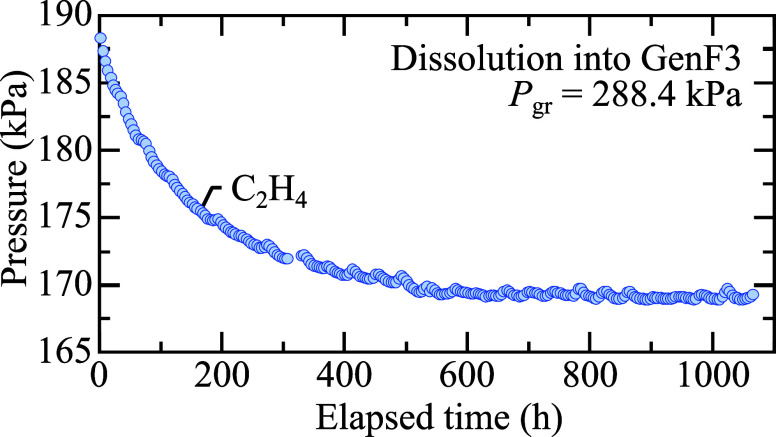
A pressure decay curve of C_2_H_4_ dissolution into GenF3 with a starting gas reservoir pressure, *P*_gr_ = 288.4 kPa. Equilibrium of the C_2_H_4_ gas pressure above the liquid and the saturated concentration of C_2_H_4_ in the liquid was reached after 1060 h at *T* = 303.15 K.

Figure 1 illustrates a long-term trial of C_2_H_4_ dissolution in GenF3 with a gas reservoir initial pressure, *P*_gr_ = 288.4 kPa. After the needle valve is opened, the gas escapes the reservoir and fills the entire cell, which causes a sharp pressure drop due to expansion of the gas into the part of the cell containing electrolyte (the headspace above the liquid). This is followed by a steep initial pressure decay, attributed to the interface-filling phenomenon as the gas saturates the topmost layers of the liquid in the reservoir.^32^ The final value *P*_eq_ is an average of the last 20 measured data points before the trial was stopped after 1060 h. The initial and equilibrium partial pressures of the gas are used to accurately calculate the change in moles moving from the gas phase to the solution phase.

**Figure 2:**
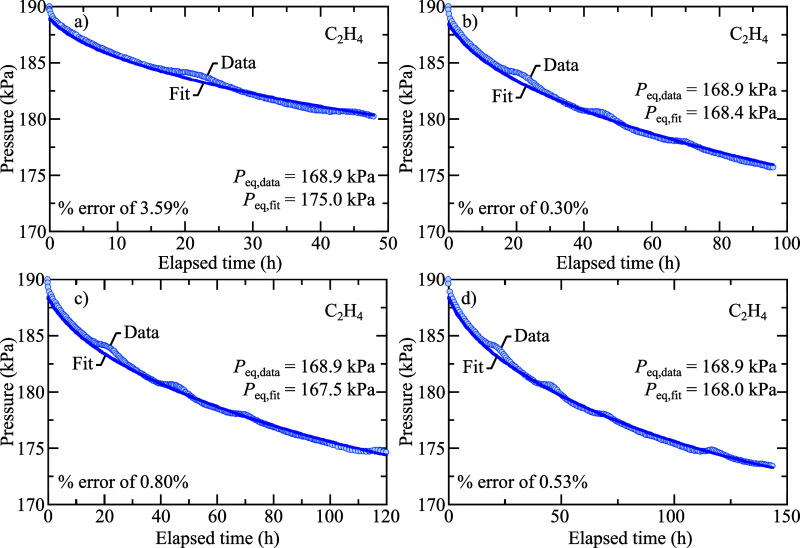
Experimental and model fit pressure decay curves of the *t* = 1060 h solubility trial of C_2_H_4_, ending at (a) 48, (b) 96, (c) 120, and (d) 144 h. Each trial produces a *P*_eq,fit_ from eq 2 that is compared to the experimental *P*_eq,data_ measured at 1060 h. From this data it was determined that at least 96 h is a sufficient amount of time for the fit to predict an accurate *P*_eq_ for C_2_H_4_ at *T* = 303.15 K.

Equation 2 was also used to extrapolate the *P*_eq_ of shorter trials (<150 h) that were concluded before equilibrium was reached. To best approximate the behavior of the slow pressure decay due to dissolution, the data sets used in the model are in seconds and begin where d*P*/d*t* < 0.034 ΔkPa s^–1^, after the interface-filling regime. To determine an optimal time for the conclusion of the trials, the *t* = 1060 h data set was used to calculate the model-fit percent error in *P*_eq_ when the trial runtime length was varied. Figure 2 shows the decreasing percent error with data sets of increasing trial runtime. After 96 h the percent error is consistently less than 1%. There is some systematic noise in Figure 2 that appear as “bumps” in the data appearing approximately 24 h apart. This systematic error is attributed to daily temperature changes in the room.

**Figure 3:**
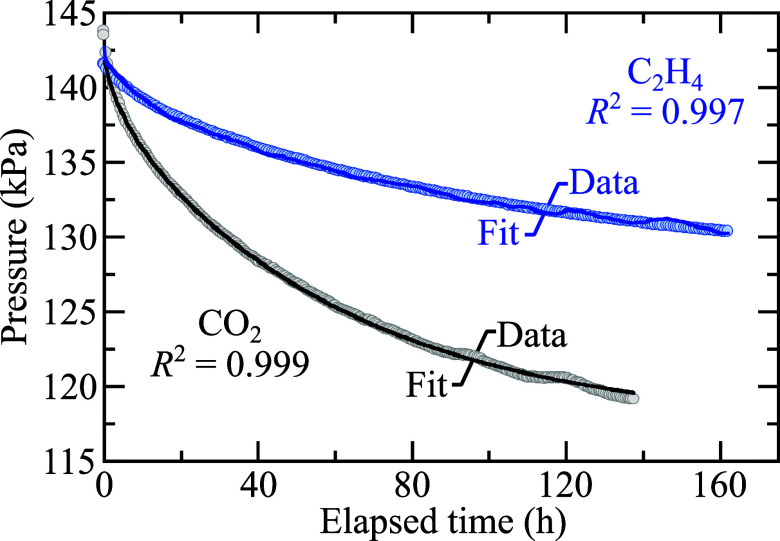
Experimental and model fit pressure decay curves with *R*^2^ comparisons for CO_2_ and C_2_H_4_ at *P*_gr_ = 193 kPa and *T* = 303.15 K.

Figure 3 shows the experimental and best-fit curves for a C_2_H_4_ and a CO_2_ trial at *P*_gr_ = 193 kPa. The fits have a coefficient of determination, *R*^2^ > 0.99, for both gases. Although the Behzadfar and Hatzikiriakos model was developed to evaluate the diffusivity of CO_2_ in bitumen,^32^ the observed *R*^2^ ≈ 1 for both gases studied here, despite large differences in decay rates, emphasizes the accuracy of the chosen model for a carbonate-based battery electrolyte.

**Figure 4:**
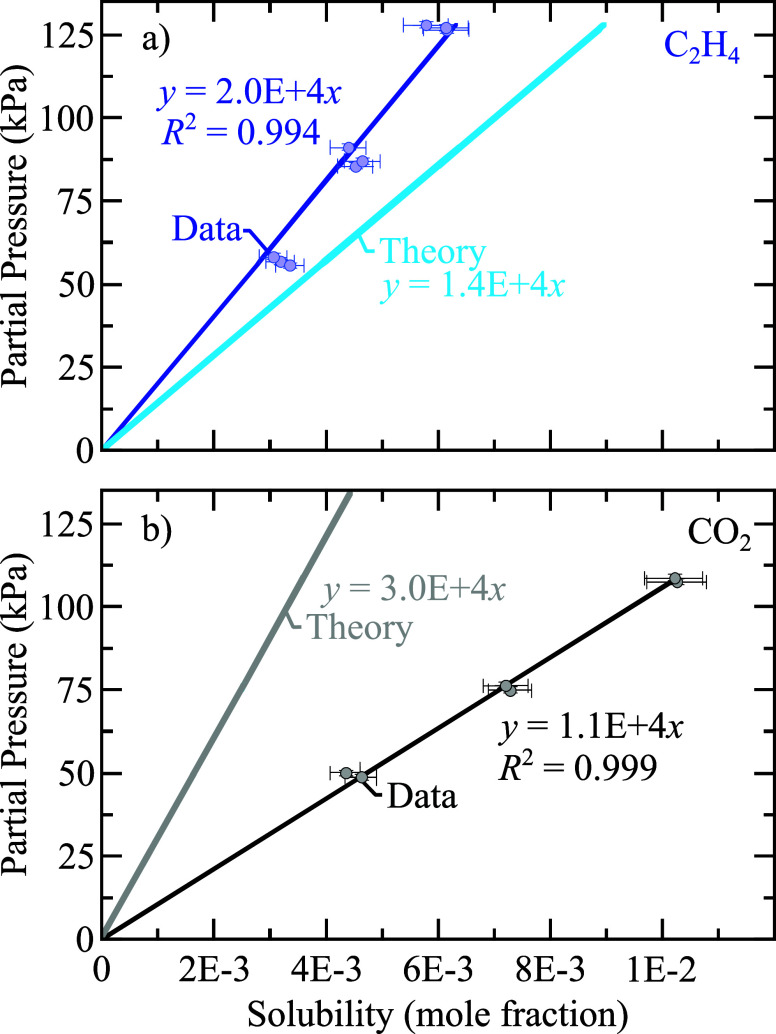
Experimental and theoretical Henry’s law curves of (a) C_2_H_4_ and (b) CO_2_ in GenF3 electrolyte at *T* = 303.15 K and 50 < *P*_eq_ < 128 kPa. The Henry’s law coefficients predicted by DFT differ from the experimental values by factors of 1.43 and 2.72 for C_2_H_4_ and CO_2_, respectively.

Figure 4 illustrates the solubility with respect to partial pressure for (a) C_2_H_4_ and (b) CO_2_. The data for the figure and their uncertainties are listed in Tables 2 and 3. The slope of the lines is the Henry’s law constant. The plots illustrate both the experimentally measured data and the DFT-predicted (labeled “theory”) trends.

**Table 2:** Measured Experimental Data Used to Calculate C_2_H_4_ in GenF3 Electrolyte Henry’s Law Constant, *k* = 2.0 × 10^4^ kPa, *u*(*k*_C_2_H_4__) = 0.1, at *T* = 303.15 K

*P*_gr_, kPa^a^	, kPa^b^	, kPa^c^	(10^3^)^d^
133.4	71.7	57.0	3.2
132.8	71.4	55.9	3.4
135.0	72.6	58.5	3.1
198.4	107	85.7	4.5
207.7	112	91.3	4.4
202.0	109	87.1	4.6
288.4	155	126.6	6.1
289.4	156	127.2	6.1
288.1	155	128.1	5.8

a C_2_H_4_ pressure of gas reservoir. Relative uncertainty *u*_r_(*P*_gr_) = 0.0009.

b Initial partial pressure of gas above the liquid, calculated from *P*_gr_. Relative uncertainty *u*_r_(*PP*_i,C_2_H_4__) = 0.009.

c Final partial pressure of gas above the liquid. Standard uncertainty *u*(*PP*_eq,C_2_H_4__) = 0.8.

d Solubility of gas in mole fraction. Relative standard uncertainty *u*_r_(χ_,C_2_H_4__) = 0.08 for *P*_gr_ = 132.8–135.0, *u*_r_(χ_,C_2_H_4__) = 0.07 for 298.4 < *P*_gr_ < 288.4.

**Table 3:** Measured Experimental Data Used to Calculate CO_2_ in GenF3 Electrolyte Henry’s Law Constant, *k* = 1.1 × 10^4^ kPa, *u*(*k*_CO_2__) = 0.04, at *T* = 303.15 K

*P*_gr_, kPa^a^	, kPa^b^	, kPa^c^	(10^3^)^d^
130.9	70.4	49.0	4.6
130.8	70.3	50.2	4.3
202.6	109	75.1	7.3
204.5	110	76.5	7.2
289.2	155	107.7	10.3
290.8	156	108.8	10.2

a CO_2_ pressure of gas reservoir. Relative uncertainty *u*_r_(*P*_gr_) = 0.0009.

b Initial partial pressure of gas above the liquid, calculated from *P*_gr_. Relative uncertainty *u*_*r*_(*PP*_i,CO_2__) = 0.009.

c Final partial pressure of gas above the liquid. Standard uncertainty *u*(*PP*_eq,CO_2__) = 0.8.

d Solubility of gas in mole fraction. Relative standard uncertainty *u*_r_(χ_,CO_2__) = 0.06 for *P*_gr_ = 130.9, 130.8, *u*_r_(χ_,CO_2__) = 0.05 for 202.6 < *P*_gr_ < 290.8.

Error analysis was conducted on the calculated equilibrium partial pressures and solubilities for the Henry’s law graphs. For the partial pressures, error is derived from the uncertainty of *P*_eq_ (±0.08%, from transducer) and subsequent subtraction of Ar partial pressure (38.61 ± 0.83 kPa). Due to a lack of data, the vapor pressure uncertainty could not be considered. For solubility, error is primarily derived from uncertainties in the initial moles, final moles, and electrolyte volume values. Specifically, since moles values are calculated assuming ideal gas behavior [*n* = *PV*/(*RT*)], error in the initial moles value includes uncertainty in the transducer pressure and the gas compartment volume measurement; error in the final moles value includes uncertainty in the total volume of the cell and the partial pressure of target gas. The electrolyte volume uncertainty is given as ±0.1 mL from the syringe manufacturer.

For the gases studied in this work, the Henry’s law coefficients predicted by DFT differ from the experimental values by factors of 1.43 and 2.72 for C_2_H_4_ and CO_2_, respectively. This discrepancy implies that the error in the predicted solvation free energy is at most 0.63 kcal mol^–1^ (0.03 eV), which is well within “chemical accuracy” (of 1 kcal mol^–1^). In part, the observed error between theory and experiment may arise because the SMD parameters used in this study to calculate *k* did not account for the effect of FEC nor the LiPF_6_ salt, which may impact the solvation free energy of small molecules. However, we attribute the error in calculated solvation free energy primarily to the fundamental limitations of implicit solvent models. As mentioned, SMD performs well on a variety of neutral small molecules in organic solvents, but it is nonetheless known to fail to capture certain effects, for instance ionic and hydrogen bonding.^45^ Calculating solvation free energies using explicit solvation shells, rather than an implicit solvent medium, may provide an opportunity to achieve better agreement with experiment.

## Discussion

The present work describes an experimental procedure to measure the solubility of gases in a commonly used lithium-ion battery electrolyte by monitoring the pressure change of a gas as it dissolves into the liquid electrolyte. The gases in this study were chosen because they readily form via (electro) chemical reactions during normal operation of a Li-ion battery during cell formation. While the time to reach equilibrium pressure and saturation gas concentration is typically 500–1000 h, it is shown here that a multiphase model allows extrapolation to equilibrium with measurements lasting <100 h. As the equilibrium constant for a given gas species is highly influenced by the electrolyte composition, including the salt concentration, it would be intractable to measure many different gas/electrolyte equilibrium concentrations without the approach described in this work.

This work also demonstrates the calculation of Henry’s law constants for these gas phase species in carbonate electrolyte, enabling the determination of their equilibrium dissolved concentrations. We anticipate that this will allow researchers to better interrogate the complex, competing reaction pathways, including gas reactants, occurring in Li-ion batteries, particularly within the SEI. Furthermore, there are relatively few Henry’s law constants reported in the literature in battery-relevant systems.^28,29^ Thus, there is an inherent need to measure and report the Henry’s law constants for predominant gas species in common Li-ion battery electrolytes.

Additionally, the thermodynamic calculations from DFT are validated by comparison to experimentally determined Henry’s law constants for the two gaseous species studied (CO_2_ and C_2_H_4_) in this work. The DFT-predicted and measured Henry’s law constants were found to agree well, with small errors ≤0.63 kcal mol^–1^. This indicates that current theoretical models can predict Henry’s law constants within an order of magnitude, potentially even without accounting for every species in solution. However, further improvements can still be made, and there is a need to develop physics-based models that can accurately determine these constants to extrapolate the present results to additional electrolyte systems.

## Conclusions

The pressure decay at different starting pressures of C_2_H_4_ and CO_2_ gas dissolving into GenF3 battery electrolyte was recorded and modeled to extrapolate the equilibrium pressures, *P*_eq_. Analysis of trial run time was conducted, determining the minimum trial length of 96 h to minimize percent error of the model fit *P*_eq_. The differences in moles from initial to equilibrium partial pressures were used to calculate the solubility of each gas at the equilibrium pressures, and this was done for several initial pressures. The equilibrium pressures versus solubility in mole fraction were plotted to calculate the Henry’s law constants *k* for each gas with  kPa and *k*_CO_2__ = 1.1 × 10^4^ kPa. Using the Environmental Protection Agency (EPA) standards, these two species would be considered “volatile” in the electrolyte studied.^46,47^ This means that the species are significantly more stable in the gas-phase as compared to the liquid-phase. In terms of studying reaction mechanisms that form SEI species, there is a significant competing pathway to eject these dissolved species to the gas-phase compared to retaining these species to feed additional reaction cascades. Nevertheless, the equilibrium saturation concentrations of both C_2_H_4_ and CO_2_ are estimated to be in the range of 0.5–1.0 mol % at reasonable partial pressures expected for a typical Li-ion battery, suggesting that an appreciable amount of gas remains in the liquid that could contribute to electrochemical side reactions during cell operation.

These experimental constants were compared to theoretical *k* constants in 3:7 wt/wt EC/EMC solution. Despite the DFT calculations not including salt coordination or FEC effects, the predicted constants differed by factors of 1.43 and 2.72 for C_2_H_4_ and CO_2_, respectively. Differences between experimental measurements and DFT calculations are attributed to limitations of the DFT solvent models and presence of additives in the EC/EMC experimental solution.

Although only two gases were measured in this study, our approach will be extended to several others—CO, C_2_H_2_, H_2_, CH_4_, and O_2_—that are typically formed during formation and cycling of a lithium-ion battery. Furthermore, additional electrolyte formulations will be explored to quantify effects on gas solubility of varying solvent, liquid additives, and salt concentrations.

## Supporting Information Available

Supporting Information that includes an annotated photo of the pressure decay apparatus used in this study, Figure S1; example of a pressure decay measurement of carbon dioxide gas in water, Figure S2, using the apparatus described in this work; and measurement of the Henry’s law constant for CO_2_ in water at *T* = 303 K, Figure S3 (PDF)
