# Supplementary material for: Solubilities of Ethylene and Carbon Dioxide Gases in Lithium-Ion Battery Electrolyte
Source: J Chem Eng Data. 2024 May 21;69(6):2236–43. doi: 10.1021/acs.jced.3c00692 (PMC11181333; doi:10.1021/acs.jced.3c00692)
Supplement: Supplementary file 1 — je3c00692_si_001.pdf [file je3c00692_si_001.pdf]

# Supporting Information for Solubilities of Ethylene and Carbon Dioxide Gases in Lithium-ion Battery Electrolyte

Mel Soto,[†] Kae Fink,[†] Christof Zweifel,[†] Peter J. Weddle,[†] Evan Walter Clark Spotte-Smith,[‡,¶] Gabriel M. Veith,[§] Kristin A. Persson,[‡,‖] Andrew M. Colclasure,[†] and Bertrand J. Tremolet de Villers*,[†]

†*National Renewable Energy Laboratory (NREL), 15013 Denver West Parkway, Golden, CO 80401, United States of America*

‡*Department of Materials Science and Engineering, University of California, Berkely, CA 94720, United States of America*

¶*Materials Science Division, Lawerence Berkeley National Laboratory, Berkeley, CA 94720, United States of America*

§*Chemical Sciences Division, Oak Ridge National Laboratory, Oak Ridge, Tennessee 37831, United States*

‖*Molecular Foundry, Lawerence Berkeley National Laboratory, Berkeley, CA 94720, United States of America*

E-mail: bertrand.tremolet@nrel.gov

## Gas solubility apparatus

Figure S1 depicts the custom apparatus designed to measure pressure decays. See Section "Pressure Decay Trials" in the main article text for details of the apparatus and the mea-

surement protocols.

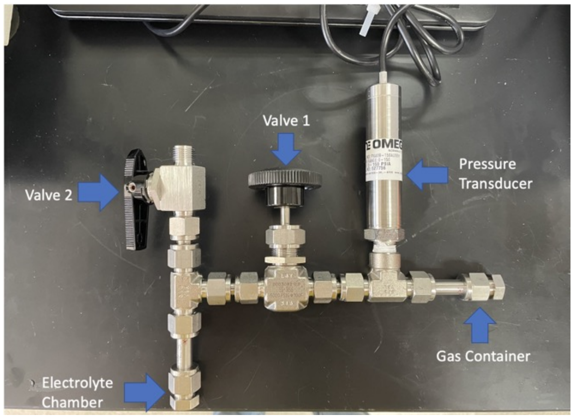


Figure S1: Swagelok solubility cell used in the pressure decay experiments. Valve 1 is the needle valve that is closed to trap gas in the gas reservoir. Valve 2 is the ball valve used to seal the entire cell after electrolyte has been added into the electrolyte chamber.

The pressures of the transuducer were reported to have uncertainties of ±0.08% according to the manufacturer.

Table S1: Volume of the Swagelok cell components and electrolyte added used for partial pressure and solubility calculations.

| Component | Volume $\pm$ $u$, mL |
|---|---|
| Empty cell | 14.78 ± 0.04 |
| Gas reservoir | 6.87 ± 0.02 |
| Electrolyte chamber | 7.91 ± 0.05 |
| Electrolyte added | 2.00 ± 0.10 |
| Empty volume after electrolyte added | 12.78 ± 0.10 |

Table S2: Pressure constants used for partial pressure and solubility calculations

| Constant | Pressure $\pm$ $u$, kPa |
|---|---|
| Argon partial pressure | 38.61 ± 0.83 |
| Electrolyte vapor pressure | 3.3 |

## Solubility of $CO_2$ in water

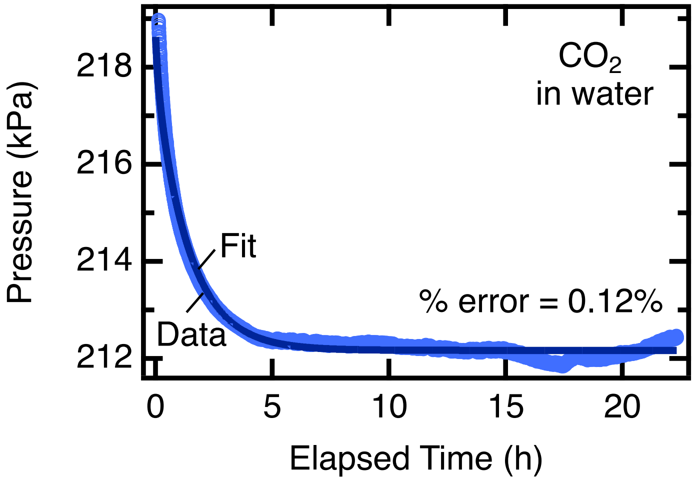


Figure S2: Experimental and model fit pressure decay curve for $CO_2$ in water at $P_{gr}$ = 384.7 kPa and T = 303.15 K.

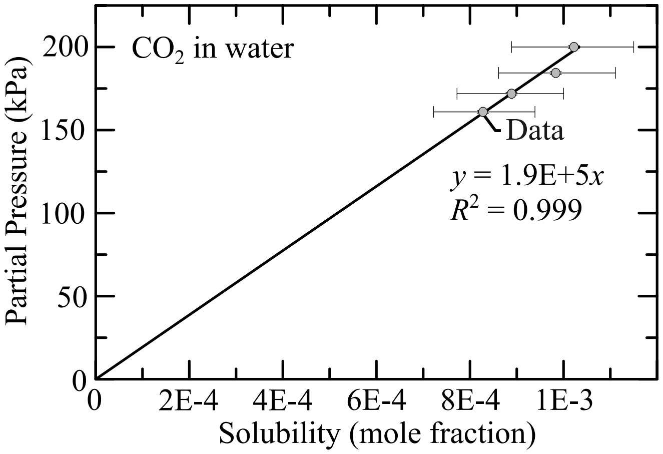


Figure S3: Henry's Law plot for $CO_2$ in water at T = 303 K. Calculated Henry's Law constant is $k_{CO_2} = 1.92 \times 10^5$ kPa, . From the IUPAC-NIST Solubility Database, $k$ of $CO_2$ in water at 303.15 K is $1.85 \times 10^5$ kPa.[1]

Table S3: Measured experimental data used to calculate **$CO_2$ in water** Henry's Law Constant, $k = 1.92 \times 10^5$ kPa, at T = 303.15 K.

| $P_{gr}$, kPa[a] | $PP_{i,CO_2}$, kPa[b] | $PP_{eq,CO_2}$, kPa[c] | $\chi_{,CO_2}$ $(\cdot 10^3)$[d] |
|---|---|---|---|
| 334.7 | 179.94 | 161.76 | 0.830 |
| 357.4 | 192.14 | 172.66 | 0.889 |
| 384.7 | 206.81 | 185.21 | 0.986 |
| 412.8 | 221.90 | 199.48 | 1.023 |

[a] $CO_2$ pressure of gas reservoir. Relative uncertainty $u_r(P_{gr}) = 0.0011$
[b] Initial partial pressure of gas above the liquid, calculated from $P_{gr}$. Relative uncertainty $u_r(PP_{i,CO_2}) = 0.012$
[c] Final partial pressure of gas above the liquid. Standard uncertainty $u(PP_{eq,CO_2}) = 0.85$
[d] Solubility of gas in mol fraction. Relative standard uncertainty $u_r(\chi_{,CO_2}) = 0.13$

Table S4: Henry's Law constant of $CO_2$ in water at T=303 K and 161 < $P_{,\ kPa}$ < 200

| $k_{CO_2}$ in water, kPa | Source |
|---|---|
| $1.92 \times 10^5$[a] | this work |
| $1.85 \times 10^5$ | IUPAC-NIST Solubility Database[1] |

[a] standard uncertainty $u(k_{CO_2}) = 0.6$

# References


(1) IUPAC-NIST Solubility Database, Version 1.1 - NIST Standard Reference Database 106. 2012; `https://srdata.nist.gov/solubility/`.
